# Enhancing Oral Absorption of an Ester Prodrug by Coating Drug Crystals with Binary Lipid Systems and Evaluating the Influence of Compositions

**DOI:** 10.3390/pharmaceutics17091210

**Published:** 2025-09-17

**Authors:** Xiaowei Dong, Tao Zhang, Hellen L. Moreno Sanchez, Jaylen C. Mans, Sung Hun Bae, Liangqiao Bian

**Affiliations:** 1 Department of Pharmaceutical Sciences, University of North Texas Health Science Center, Fort Worth, TX 76107, USA; 2 Department of Pharmaceutical Sciences, SUNY-Binghamton University, Binghamton, NY 13902, USA; zhangt@binghamton.edu (T.Z.);; 3 Shimadzu Center for Advanced Analytical Chemistry, University of Texas at Arlington, Arlington, TX 76019, USA

**Keywords:** ester prodrugs, degradation, stability, olmesartan medoxomil, oral absorption enhancement

## Abstract

**Background/Objectives**: Prodrug strategies are a vital aspect of drug development, with ester prodrugs particularly notable for modifying parent drug properties through ester functional groups to enhance oral absorption. However, ester prodrugs are prone to hydrolysis by water and enzymes, making stability in the gastrointestinal (GI) tract prior to absorption a key challenge. Few formulation strategies effectively address this degradation issue. We recently introduced binary lipid systems (BLS), comprising a lipid and a water-soluble surfactant only that form stable microemulsions. This study aimed to explore the application of BLS for enhancing the oral absorption of ester prodrugs by coating drug crystals with BLS in solid granules and study the impact of the compositions of BLS on oral absorption. **Methods**: Olmesartan medoxomil (OLM), a methyl ester prodrug of olmesartan (OL), was selected as a model drug. Various lipids were combined with TPGS to form BLS and used to prepare OLM solid granules containing OLM crystals. **Results**: Among the tested formulations, OLM MCM-TPGS granules significantly enhanced drug release and protected OLM from enzyme-mediated degradation in two-step dissolution studies with esterase. Pharmacokinetic and tissue distribution studies in rats confirmed that OLM MCM-TPGS granules improved oral absorption by 145% and increased tissue uptake compared to OLM powder. **Conclusions**: This approach overcomes solubility limitations when using lipids and surfactants as excipients, enabling high drug loading in solid dosage forms and expanding the utility of lipids and surfactants for water-insoluble drugs. This novel formulation strategy holds great potential for enhancing oral absorption of ester prodrugs, representing a significant advancement in formulation technologies and offering more effective and versatile drug delivery solutions.

## 1. Introduction

Prodrug strategies have emerged as a vital aspect of drug discovery and development, frequently employed to address deficiencies in drug properties by improving solubility, permeability, stability, taste, and gastrointestinal (GI) tolerance [1,2,3,4]. Prodrugs themselves are pharmacologically inactive but are converted into the active parent drugs in vivo through enzymatic and/or chemical reactions. Notably, nearly 10% of all FDA-approved drugs are prodrugs. Over the past decade, the FDA has approved approximately 50 prodrugs, accounting for 13% of all approved small-molecule new chemical entities [5].

Among various prodrug strategies, ester prodrugs are particularly notable for their ability to modify the pharmacokinetic (PK) and pharmacodynamic properties of parent drugs by introducing ester functional groups [6]. Ester prodrugs commonly enhance the lipophilicity of parent drugs by masking polar functionalities, such as alcohol or carboxylic acid groups, converting them into esters and thereby improving membrane permeability. Examples of ester prodrugs include simple esters, amino acid esters, sugar esters, lipid esters, polymeric esters, thioesters, and phosphoesters. These approaches are widely used to modulate the solubility and permeability of parent drugs [2,4]. However, achieving a balance between solubility and permeability can be challenging. While masking polar groups of the parent drug increases lipophilicity, it can also reduce solubility. Additionally, ester prodrugs are often susceptible to aqueous and enzymatic hydrolysis in the GI tract, especially in cases where hydrolysis is catalyzed by varying pH conditions within the GI tract. Esterases, which catalyze the hydrolysis of esters into carboxylic acids and alcohols, are responsible for breaking down a wide range of chemical bonds, including esters, thioesters, phosphoesters, amides, and epoxides [6,7]. The stability of ester prodrugs in the GI tract is therefore crucial to maximize target-site exposure of the parent drugs [4]. Whether intended to enhance solubility or permeability, oral ester prodrugs must possess sufficient stability in the GI tract and enterocytes to avoid premature hydrolysis, enabling them to reach the bloodstream and liver where they are converted to their active forms. Ester prodrugs can enhance oral bioavailability only if they remain stable within the GI tract. Despite considerable efforts to improve their stability, most solutions have primarily focused on chemical modifications to the structures of ester prodrugs. Recent advances in nanoparticle technology have shown promise in protecting ester prodrugs from aqueous environments by encapsulating them within nanoparticles [8]. However, beyond nanoparticle encapsulation, formulation-based strategies have been largely underexplored as potential solutions to these challenges.

Recently, we investigated and demonstrated a novel class of lipid-based drug delivery system, termed binary lipid systems (BLS). These systems consist of a single lipid and a single water-soluble surfactant, which together form stable microemulsions [9]. We developed and explored prototypes of BLS using various lipids and surfactants. To assess the potential of BLS for oral solid dosage forms, we also investigated solid microemulsions, in which the BLS is absorbed onto solid carriers to produce solid granules that form stable microemulsions upon contact with aqueous media. In this study, we evaluated the application of BLS in solid granules to improve the oral absorption of ester prodrugs and studied the impact of the compositions of BLS on oral absorption. Conventional lipid-based formulations, such as emulsions, microemulsions, and lipid nanoparticles, require the drug to be fully solubilized in excipients, which limits drug loading capacity [10,11]. Additionally, these formulations are typically prepared as liquid dosage forms, which pose stability challenges [12]. To address these issues, we explored a novel approach of creating solid drug granules by coating drug crystals with BLS, thereby resolving the challenges of low drug loading and stability. Lipids have diverse metabolic pathways and functional properties. For instance, long-chain triglycerides are digested by enzymes in the stomach and small intestine into mono- and diglycerides, stimulating lipoprotein formation and facilitating lymphatic transport. In contrast, medium-chain monoglycerides are either directly transported to systemic circulation via the portal blood or further digested in the small intestine by carboxylesterases (CE) that are esterases to hydrolyze carboxylic esters [13]. Additionally, lipids have demonstrated potential to enhance drug permeability [14,15]. Based on these properties, we hypothesize that selecting appropriate lipids for the BLS can protect ester prodrugs from degradation in the GI tract and enhance their solubility and permeability, ultimately leading to improved oral absorption.

The objective of this study is to explore the application of BLS for enhancing the oral absorption of ester prodrugs by coating drug crystals with BLS and study the impact of the compositions of BLS on oral absorption. Olmesartan medoxomil (OLM), a selective angiotensin II receptor blocker approved for the treatment of hypertension [16], was chosen as the model drug. OLM is a methyl ester prodrug of olmesartan (OL). OL is di-anionic due to its carboxyl (pKa 3.7) and tetrazole (pKa 4.8) groups, leading to complete ionization in the GI tract, which makes OL poorly permeable. To mask these acidic groups and improve permeability, a prodrug approach has been used for OL by esterifying the carboxyl group into a mono-anionic methyl ester, resulting in OLM [17]. Once absorbed, OLM is rapidly and completely converted to OL in the blood and liver before entering systemic circulation [18]. However, this increase in lipophilicity simultaneously reduces solubility, making OLM poorly soluble in water (~10 µg/mL). Additionally, OLM undergoes hydrolysis in the GI tract, converting it into the impermeable parent drug OL through unfavorable ester bond cleavage by water and CE [19,20]. OLM also experiences P-glycoprotein-mediated efflux, further complicating its absorption [21]. These challenges contribute to OLM’s low oral bioavailability (~26%) [22], making it a suitable model drug to test the hypothesis of this study. OLM solid granules were prepared using BLS with different lipids and evaluated through in vitro two-step dissolution studies and in vivo PK and tissue distribution studies. To examine the protective effect of BLS against CE-mediated hydrolysis, CE was added to the dissolution medium. Six OLM formulations were developed and compared to assess their ability to enhance oral absorption and to elucidate the mechanisms by which BLS provides protection for OLM.

## 2. Methods and Materials

### 2.1. Materials

Migloyl 812 was generously provided by Cremer (Eatontown, NJ, USA). Capmul MCM C8 and propylene glycol monocaprylate (Capmul PG-8) were kindly supplied by Abitec (Columbus, OH, USA). Aeropearl 300 and Avicel 105 were obtained as gifts from Evonik (Parsippany, NJ, USA). D-α-tocopheryl polyethylene glycol 1000 succinate (TPGS) was provided as a gift by Antares (Saint Charles, IL, USA). OLM was procured from TCI America (Portland, OR, USA), and OL was sourced from Santa Cruz Biotechnology (Dallas, TX, USA). Acetonitrile and methanol were purchased from Fisher Scientific (Pittsburgh, PA, USA), while phosphoric acid (H_3_PO_4_) was obtained from Sigma-Aldrich (St. Louis, MO, USA). Esterase from porcine liver, a source of CE, was purchased from Sigma-Aldrich (St. Louis, MO, USA).

### 2.2. Animals

Sprague-Dawley rats (male, 250–300 g) were purchased from Charles River Laboratories (Wilmington, MA, USA). All animal experiments were performed in compliance with a protocol approved by the Institutional Animal Care and Use Committee (IACUC) at the University of North Texas Health Science Center. The rats were housed in pairs under a 12 h light/dark cycle with free access to food and water for one week prior to the experiments.

### 2.3. Selection of Lipid and Surfactant for Construction of Binary Lipid Systems

TPGS, a surfactant and a synthetic derivative of vitamin E, was selected as the surfactant in the BLS. Pharmaceutical lipids including glycerides such as Capmul MCM C8 and Miglyol 812 and propylene glycol such as Capmul PG-8, which are approved by the FDA, were chosen to form the BLS with TPGS. The chemical compositions of lipids used in the study are shown in Table 1.

### 2.4. HPLC Method for OLM Measurement

An HPLC method to simultaneously measure both OL and OLM was conducted as previously reported [23]. Briefly, a Waters HPLC system equipped with an Eclipse XDB C18 column (3.5 µm, 4.6 × 100 mm; Agilent, Santa Clara, CA, USA) was used. The flow rate was set at 1.0 mL/min, and the mobile phase consisted of solvent A (pure methanol) and solvent B (0.1% H_3_PO_4_ in water). The injection volume was 20 µL. A gradient elution was employed with the following program: solvent A was maintained at 50% for 2 min, increased to 60% by 4 min, returned to 50% at 6.1 min, and held at 50% until 8.5 min. For sample preparation, a mixture of solvent A and solvent B in a 1:1 (*v*/*v*) ratio was used as the mobile phase for sample dilution as described below.

### 2.5. Preparation of OLM Formulations

The compositions of OLM formulations are shown in Table 2. Briefly, OLM, lipid and TPGS were mixed at 37 °C for 15 min with stirring, and then Aeroperl 300 was added and mixed. After cooling to room temperature, OLM granules were formed. OLM was directly mixed with Avicel 105 to prepare OLM powder. Drug loading was calculated as (OLM weight)/(total formulation weight) × 100%.

### 2.6. Particle Size Measurement of Reconstituted OLM Formulations

To evaluate particles generated from OLM formulations, 1 mL MilliQ water was added into 10 mg OLM granules or 5 mg OLM MCM-TPGS Liquid. After mixed and centrifugation, particle size and size distribution in the supernatants were measured by dynamic light scattering using a particle analyzer (Malvern Zetasizer Ultra Particle Analyzer, Malvern Panalytical, Westborough, MA, USA). The polydispersity index (PI) was used to indicate size distribution.

### 2.7. Determination of Physical State of OLM in OLM Formulations

The physical state of OLM within OLM formulations was evaluated visual observation during the formulation preparation and measured by powder X-ray diffraction (XRD). XRD was performed using a Bruker D8 Advance (Bruker, Karlsruhe, Germany) to determine the physical state of OLM in the formulations. The measurements were conducted over a 2θ range of 8–25° with a scan rate of 1.0 s per step.

### 2.8. Two-Step Dissolutions of OLM Formulations in the Absence and Presence of an Esterase

Two two-step dissolution studies were performed as previously reported with modification [24] to evaluate the release of OLM from its formulation and elucidate the mechanisms underlying absorption enhancement. Since the pH conditions in the human GI tract differ from those in rats, two sets of dissolution media were employed. The first set consisted of simulated gastric fluid (SGF, pH 1.2) followed by simulated intestinal fluid (SIF, pH 6.8), mimicking the pH conditions of the human stomach and intestine [24]. The second set used SGF at pH 3.5 and followed by SIF at pH 6 to replicate the GI environment in rats. Additionally, to assess the impact of enzyme-mediated hydrolysis on OLM, dissolution studies were conducted both in the absence and presence of CE, an esterase responsible for hydrolyzing OLM as well as mono- and di-glycerides in the small intestine and cells [13,19]. Because CE is present in the small intestine, CE was added into the second step of dissolution in pH 6.8 (human) or pH 6 (rat).

For the dissolution studies conducted in the absence of CE, the procedures were performed to mimic human and rat GI conditions. To mimic the human GI conditions, OLM formulation (50 mg) was added into 4.5 mL SGF at pH 1.2 at 37 °C stirred at 100 rpm. Samples of 200 µL were collected at 15, 30, 45, and 60 min, and replaced with fresh medium after each collection. At 60 min, 4.5 mL of KH_2_PO_4_ (99.9 mM) and 250 µL NaOH (2M) were added to adjust the medium to SIF at pH 6.8. At 65, 90, 120, 150, and 180 min, 200 µL samples were withdrawn and replaced with fresh medium. To mimic the rat GI conditions, OLM formulation (50 mg) was added into 5 mL SGF at pH 3.5 at 37 °C stirred at 100 rpm. At 15, 30, 45, and 60 min, 200 µL samples were collected and replaced with fresh medium after each collection. At 60 min, 150 µL NaOH (2M) were added into to adjust the medium to SIF at pH 6. At 65, 90, 120, 150, and 180 min, 200 µL samples were withdrawn and replaced with fresh medium. For both conditions, collected samples were centrifuged at 15,000 rpm for 10 min, and the supernatants were diluted with the HPLC mobile phase at a 1:10 ratio. The diluted samples were analyzed using HPLC.

For the dissolution studies conducted in the presence of CE, the same procedure was followed with modification. At 65 min, after sample collections, 10 mg of CE were added to produce a final enzyme concentration of 2 mg/mL. Then samples were collected at 75, 90, 120, 150, and 180 min. The collected samples were centrifuged at 15,000 rpm for 10 min. The supernatants were then mixed with methanol at a 1:1 ratio (*v*/*v*) and then centrifuged again to remove precipitated CE. The final supernatants were diluted with the HPLC mobile phase at a 1:10 ratio (*v*/*v*) before analysis using HPLC. All experiments were performed in triplicate.

### 2.9. Pharmacokinetics of OLM Formulations in Rats

PK of OLM formulations were evaluated in male Sprague-Dawley rats (*n* = 4–6 per group). The rats were randomly assigned to groups and administered OLM formulations by oral gavage at a dose of 2 mg/kg OLM. Prior to dosing, OLM formulations were mixed with water to create suspensions. Blood samples were collected at 0, 0.5, 1, 2, 3, 4, 5, 6, 8, 10, 12, and 24 h post-dosing in heparin-coated tubes and immediately centrifuged at 4000 rpm for 5 min at 4 °C to separate plasma. Plasma samples were stored at −80 °C until analysis.

Because OLM is rapidly and completely converted to OL in the body and only OL is detectable in the body [18], OL concentrations in plasma and tissue samples were measured using a validated LC-MS method. Plasma samples were mixed with paclitaxel (internal standard) and extracted with a methanol-acetonitrile solution (1:3, *v*/*v*) containing 0.1% formic acid. The mixture was shaken at room temperature for 30 min, followed by centrifugation at 15,000 rpm for 10 min. The supernatants were analyzed using an LC-MS/MS system (Shimadzu LCMS-8060 triple quadrupole mass spectrometer). The LC system included pumps A and B (LC-30AD) and an autosampler (SIL-30AC, Shimadzu Scientific Instruments, Tokyo, Japan). The analysis was performed in positive electrospray ionization mode (+ESI) with multiple reaction monitoring (MRM). Instrument parameters were optimized as follows: interface voltage at 4.0 kV, interface temperature at 300 °C, DL temperature at 300 °C, heating block temperature at 400 °C, drying gas (N_2_) at 10 L/min, nebulizing gas (N_2_) at 3 L/min, heating gas (air) at 10 L/min, and collision-induced dissociation (CID) gas (Ar) at 230 psi. Transitions for OLM (906.5+ > 743.4+) and the internal standard (876.4+ > 308.2+) were selected based on method optimization, ensuring optimal MRM responses. Data were processed using Shimadzu LabSolutions software (v5.99). The LC mobile phases consisted of (A) 0.1% formic acid in water and (B) 0.1% formic acid in acetonitrile. The gradient program progressed from 10% to 98% B over 2 min, held at 98% B for 1.2 min, and equilibrated at 10% B for 1.3 min. The flow rate was set to 0.5 mL/min with an injection volume of 5 µL. The LC-MS method was validated for linearity, precision, accuracy, carryover, and specificity as previously described [25].

PK parameters were evaluated using non-compartmental analysis conducted with Phoenix WinNonlin software (version 8.40, Princeton, NJ, USA). The parameters included peak plasma concentration (C_max_), terminal elimination half-life (T_1/2_), time to peak concentration (T_max_), area under the plasma concentration-time curve from time 0 to the last measurable concentration (AUC_last_), area under the plasma concentration-time curve from time 0 to infinity (AUC_inf_), apparent volume of distribution (V_z_/F), and apparent total body clearance (CL/F).

### 2.10. Tissue Distribution of OLM Formulations in Rats

The tissue distribution of OLM formulations was assessed in male Sprague-Dawley rats (*n* = 4 per group) using a validated LC-MS method. Male rats (250–300 g) were randomly assigned to groups and administered OLM formulations at a dose of 2 mg/kg. After 2 h, the rats were sacrificed, and tissues including plasma, lung, liver, kidney, brain, heart, spleen, and mesenteric lymph nodes were collected. Tissue samples were stored at −80 °C until analysis. The collected tissues were weighed and homogenized in a methanol-water mixture (70:30, *v*/*v*) containing 0.1% formic acid. Homogenates were processed using protein precipitation and extraction methods as described above. The OL concentrations in the tissue samples were quantified using the validated LC-MS method described above.

### 2.11. Statistical Analysis

Data are presented as mean ± standard deviation (SD). Comparisons between two groups were made using an unpaired Student’s *t*-test. Statistical comparisons among the six groups were performed by one-way analysis of variance (ANOVA) using Graphpad Prism (version 10, San Diego, CA, USA). A *p*-value of <0.05 was considered statistically significant.

## 3. Results

### 3.1. Excipient Selection for Binary Lipid Systems in the Study

In previous studies, we found that TPGS, compared to Tween 80 and Cremophor EL, is more effective in forming stable particles with dispersed lipid droplets [9]. Moreover, TPGS inhibits the function of P-glycoprotein, preventing P-glycoprotein-mediated efflux [26]. Therefore, TPGS was selected as the surfactant for the BLS formulations tested in this study. Propylene glycol and glycerides are commonly used pharmaceutical lipids. Medium-chain glycerides, which are not digestible in the GI tract, could provide protective effects for OLM. Consequently, medium-chain lipids were chosen to form the BLS. The chemical compositions of the lipids used in this study are listed in Table 1. These excipients are approved by the FDA for oral administration. Capmul MCM C8 represents medium-chain mono- and diglycerides, Miglyol 812 represents medium-chain triglycerides, and Capmul PG-8 was selected as a representative of medium-chain propylene glycol. Aeroperl 300 was used as the solid carrier to form solid granules. When the selected lipid and TPGS were used in ratios between 7:3 and 4:6, the resulting solid granules produced stable microemulsions upon contact with water [9].

### 3.2. OLM Formulation Preparation

OLM granules and OLM powder were designed to contain about 10% drug loading. The compositions of the OLM formulations are shown in Table 2. OLM powder was prepared as a control. OLM solid granules were formulated using Capmul MCM C8, Miglyol 812, and Capmul PG-8. To investigate the influence of individual components on the OLM MCM-TPGS granules, two additional formulations were prepared: OLM MCM granules (without TPGS) and OLM MCM-TPGS Liquid (without Aeroperl 300).

The solubility of OLM is relatively low in the selected excipients: Capmul MCM C8 (~7 mg/g), Miglyol 812 (<1 mg/g), Capmul PG-8 (~11 mg/g), and TPGS (~8 mg/g). At 10% drug loading, the excipients in the formulations were unable to fully solubilize OLM. TPGS melted at 37 °C and mixed well with the lipid to form the BLS, which was subsequently coated onto OLM crystals. After adding Aeroperl 300 and cooling to room temperature, solid granules with good flowability were successfully prepared. Upon contact with water, OLM granules and OLM MCM-TPGS Liquid produced particles with size in the range of 110–250 nm with narrow size distribution.

### 3.3. Characterization of OLM Crystals in OLM Formulations

**Figure 1:**
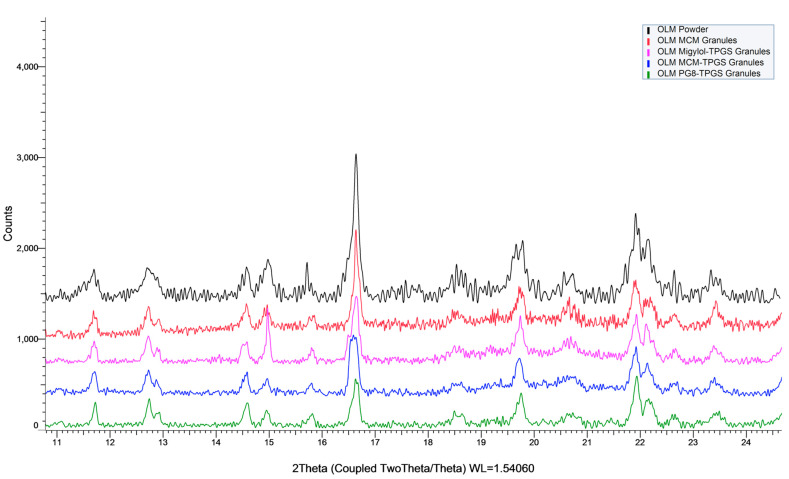
The XRD measurements of OLM formulations. OLM was confirmed to be present in its crystalline form in the formulations, as evidenced by OLM crystal peaks in the XRD measurements.

During the preparation of OLM granules, OLM crystals did not dissolve in the excipients and were clearly visible to the naked eye. The lipid and TPGS formed a homogeneous mixture that coated the surface of the OLM crystals. According to the XRD measurement (Figure 1), OLM existed as its crystal form in all samples, confirming that OLM formulations contained OLM crystals. The XRD results confirmed the presence of OLM crystals in the formulations. (Figure 1).

### 3.4. Two-Step Dissolution Mimicking Human GI Conditions in the Absence and Presence of Carboxylesterase

**Figure 2:**
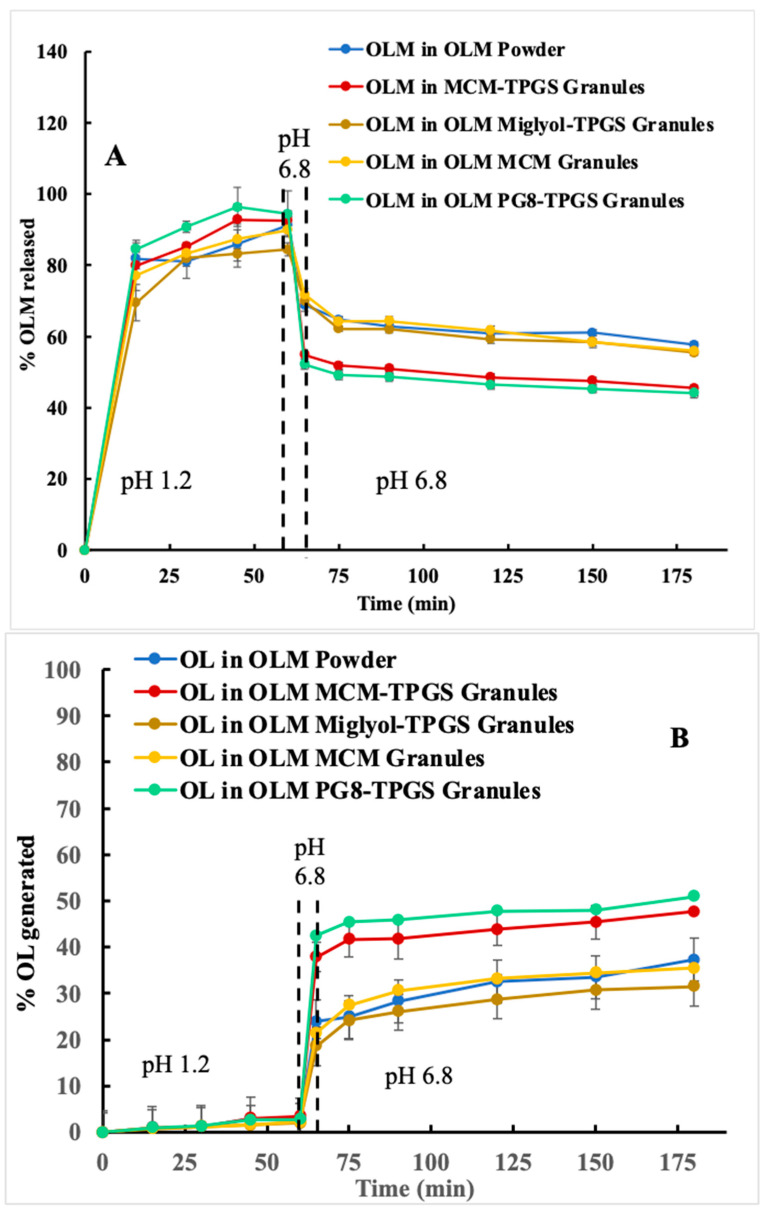

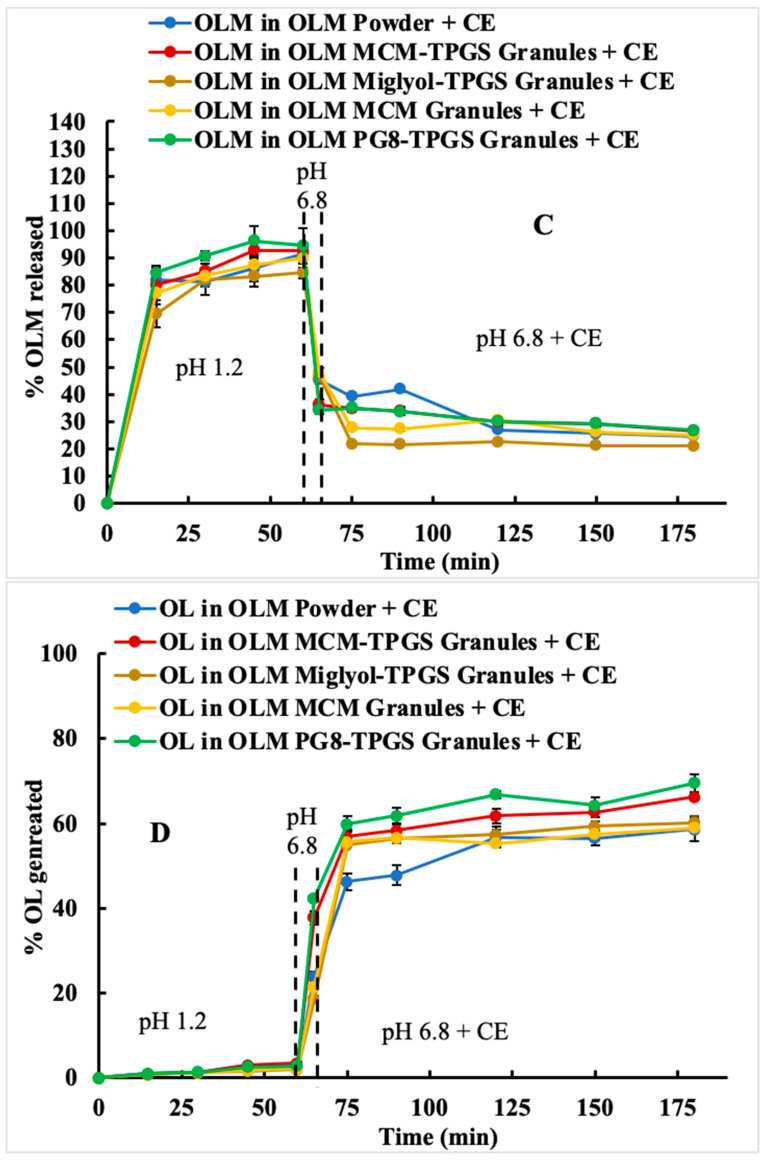
Two-step dissolution of OLM formulations in SGF (pH 1.2) and SIF (pH 6.8) to mimic the human GI conditions in the absence and presence of carboxylesterase (CE) (*n* = 3). pH was changed at 60 min from 1.2 to 6.8. CE was added at 65 min. OLM (ester prodrug) and OL (parent drug) were measured simultaneously by HPLC. (**A**) OLM release in pH 1.2 and 6.8. (**B**) OL production in pH 1.2 and 6.8. (**C**) OLM release in pH 1.2 and 6.8 in the presence of CE. (**D**) OL production in pH 1.2 and 6.8 in the presence of CE.

OLM has pH-dependent solubility with a solubility of about 825 µg/mL at pH 1.2 and 10 µg/mL at pH 6 [23]. As shown in Figure 2A, OLM release from the formulations reached approximately 100% within 60 min at pH 1.2. However, after the pH was changed to 6.8, OLM release decreased below 60% at 180 min, indicating precipitation of dissolved OLM at pH 6.8. For OL production, the OLM formulations generated less than 3% OL within 60 min at pH 1.2 (Figure 2B) despite high OLM concentrations in pH 1.2 (Figure 2A). After the pH was adjusted to 6.8, OL production significantly increased to 25–45% (Figure 2B), indicating OLM aqueous hydrolysis, which was more pronounced at pH 6.8 compared to pH 1.2 [23]. Without CE, OLM MCM-TPGS granules and OLM PG8-TPGS granules showed lower OLM concentrations and higher OL production in pH 6.8 among the tested formulations.

In the presence of CE, OLM concentrations decreased over 40% and OL concentration increased over 20% compared to the release without CE (Figure 2A,B), demonstrating CE-mediated hydrolysis. However, OLM MCM-TPGS granules and OLM PG8-TPGS granules exhibited a smaller decrease in OLM and correspondingly lower OL production compared to OLM powder, OLM Miglyol-TPGS granules, and OLM MCM granules (Figure 2C,D). These results suggest that OLM MCM-TPGS granules and OLM PG8-TPGS granules provided protection against CE-mediated degradation of OLM.

### 3.5. Two-Step Dissolution Mimicking Rat GI Conditions in the Absence and Presence of CE

**Figure 3:**
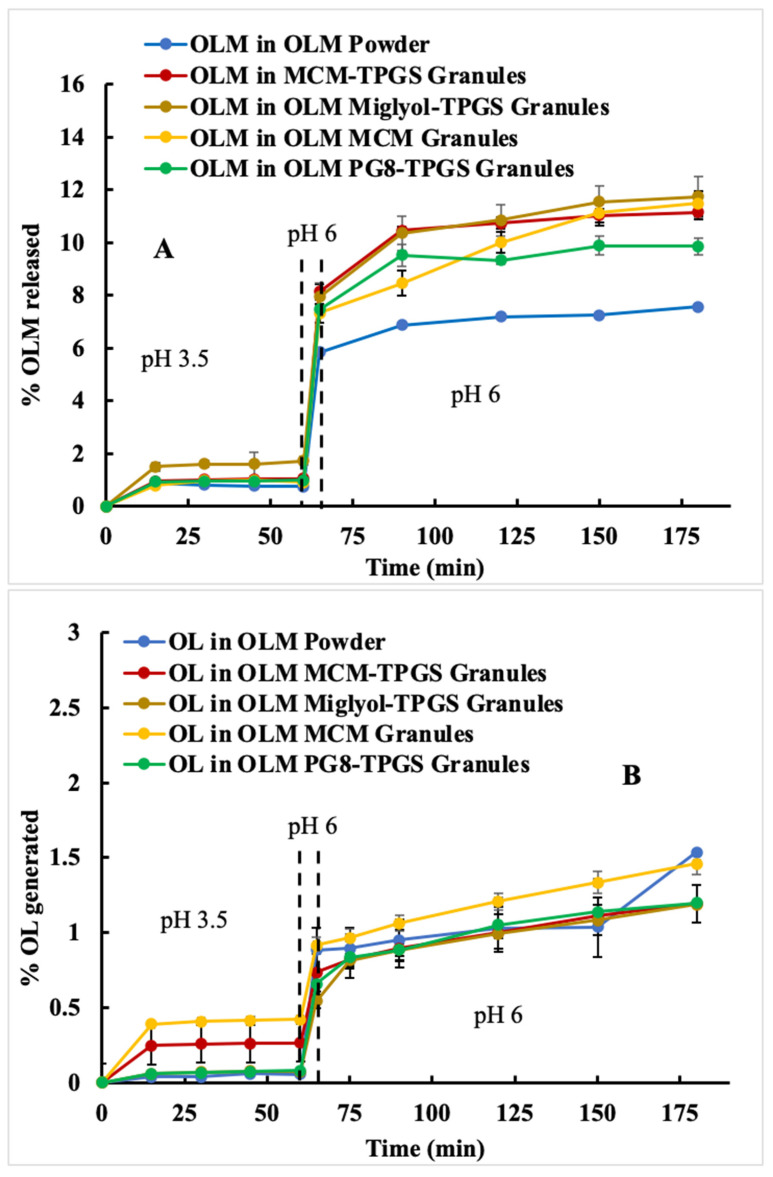

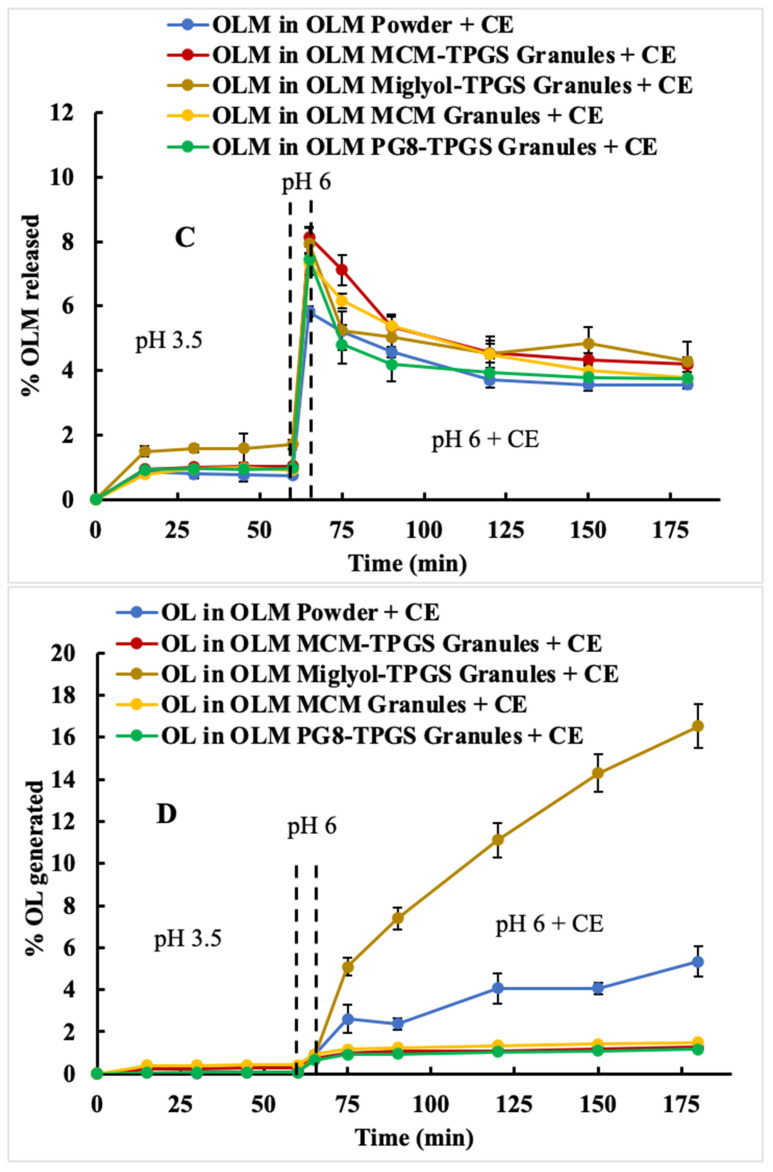
Two-step dissolution of OLM formulations in pH 3.5 and pH 6 to mimic the rat GI conditions in the absence and presence of carboxylesterase (CE) (*n* = 3). pH was changed at 60 min from 3.5 to 6. CE was added at 65 min. OLM (ester prodrug) and OL (parent drug) were measured simultaneously by HPLC. (**A**) OLM release in pH 3.5 and 6. (**B**) OL production in pH 3.5 and 6. (**C**) OLM release in pH 3.5 and 6 in the presence of CE. (**D**) OL production in pH 3.5 and 6 in the presence of CE.

The pH in the rat stomach is approximately 3.2 (fed) and 3.9 (fasted), while the pH in the rat small intestine is around 6 [27]. PK studies were conducted in rats; therefore, a two-step dissolution at pH 3.5 and pH 6 was performed to better correlate with the PK profiles of the OLM formulations evaluated in rats. OLM exhibits pH-dependent solubility, with a solubility of approximately 3 µg/mL at pH 3.5 and 10 µg/mL at pH 6 [23]. In the absence of CE, at pH 3.5, less than 2% of OLM was released from the formulations, but the release increased to 6–8% when the pH was changed to 6 at 60 min and continued to rise to about 10%, except for OLM powder (Figure 3A). OLM powder showed the lowest dissolution at pH 6, indicating OLM granules enhanced OLM dissolution at pH 6. Less than 1.5% of OL were produced at pH 6, indicating low aqueous hydrolysis in this condition (Figure 3B).

After the addition of CE at pH 6 at 65 min, there was a decrease in OLM due to OL conversion. However, OLM Miglyol-TPGS granules displayed a rapid drop in OLM release but a high increase in OL production, which indicated poor protection against enzymatic degradation (Figure 3C,D). In contrast, OLM MCM-TPGS granules and OLM PG8-TPGS granules both displayed a more gradual decline in OLM release and less OL production over time, which suggested better protection against enzymatic hydrolysis (Figure 3C,D).

### 3.6. Pharmacokinetics of OLM Formulations

**Figure 4:**
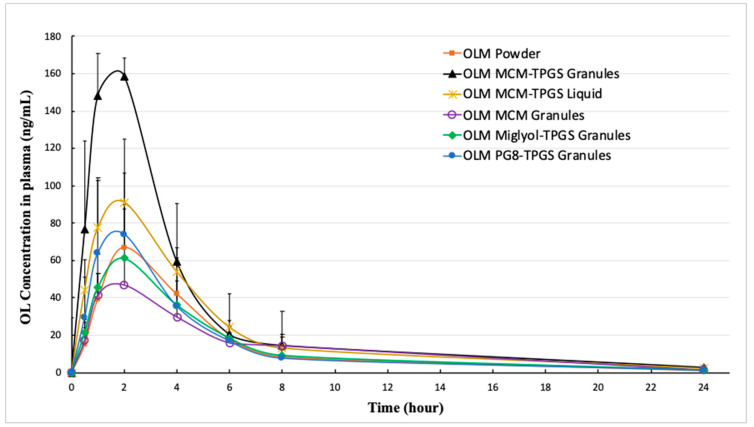
The concentration-time profiles of OL in plasma after oral administration at a dose of 2 mg/kg OLM from OLM powder (*n* = 6), OLM MCM-TPGS granules (*n* = 4), OLM MCM-TPGS Liquid (*n* = 5), OLM MCM granules (*n* = 4), OLM Miglyol-TPGS granules (*n* = 5), and OLM PG8-TPGS granules (*n* = 5) to rats, respectively. OL concentrations in plasmas were measured by a validated LC-MS method.

Since OLM was rapidly and fully metabolized to OL in the body, the plasma concentration of OL was measured for analysis. The plasma concentration-time profiles of OL following oral administration of OLM at a dose of 2 mg/kg in rats are presented for six different formulations (powder, MCM-TPGS granules, MCM-TPGS Liquid, M812-TPGS granules, MCM granules, and PG8-TPGS granules) in Figure 4. The corresponding PK parameters are summarized in Table 3. Compared to OLM powder, the C_max_ of OLM MCM-TPGS granules, OLM MCM-TPGS Liquid and OLM PG8-TPGS granules was increased by 149%, 43% and 13%, respectively, whereas OLM Miglyol-TPGS granules and OLM MCM granules showed no improvement. For T_max_, all formulations demonstrated similar times to reach peak concentrations, ranging from 1.60 h to 2.00 h, with no statistically significant differences (*p* < 0.05). The Vz/F and CL/F values of OLM MCM-TPGS granules were lower compared to other formulations, though the differences were not statistically significant except for a reduction in CL/F compared to OLM MCM granule. In terms of systemic exposure, the AUC_last_ and AUC_inf_ for OLM MCM-TPGS granules were significantly higher than those for OLM powder, with increases of 105% and 145%, respectively. These data indicate that the OLM MCM-TPGS granules significantly enhanced absorption compared to OLM powder. However, OLM MCM-TPGS Liquid and OLM MCM granules did not increase absorption compared to OLM powder, highlighting the importance of the BLS composed of Capmul MCM C8 and TPGS and the solid granule form in facilitating absorption enhancement. OLM Miglyol-TPGS granules and OLM PG8-TPGS granules also did not increase oral absorption compared to OLM powder, demonstrating that Capmul MCM C8 (mono- and di-glycerides) is superior to Miglyol 812 (triglycerides) and Capmul PG-8 (propylene glycol) for enhancing oral absorption. Additionally, OLM MCM granules exhibited the lowest absorption compared to the formulations containing TPGS, demonstrating TPGS’s role in enhancing OLM oral absorption, likely by inhibiting P-glycoprotein efflux [26]. In summary, the OLM MCM-TPGS granule formulation provided the highest systemic exposure and bioavailability, with significantly improved C_max_ and AUC values compared to the other tested formulations.

### 3.7. Tissue Distribution of OLM Formulations

**Figure 5:**
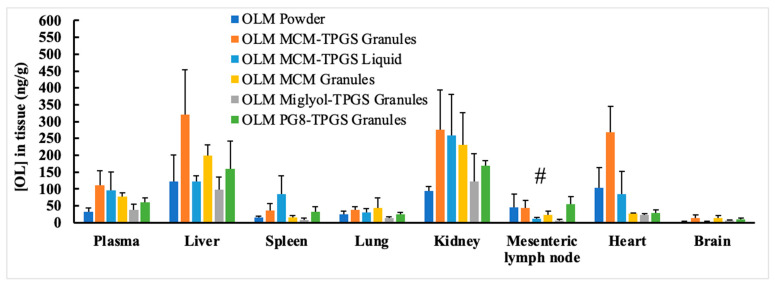
Tissue distribution of the six tested OLM formulations (*n* = 4). Rats were dosed with OLM formulations at 2 mg/kg by oral administration. Tissues were collected 2 h posting dosing, and OL concentrations in the tissues were measured by a validated LC-MS method. # *p* > 0.05 within the group by ANOVA.

Tissue distributions of OLM formulations were measured in rats after 2 h post dosing at 2 mg/kg OLM. As shown in Figure 5, OLM MCM-TPGS granules increased the uptake in liver and heart compared to other formulations. OLM MCM-TPGS Liquid increased the uptake in spleen compared to other formulations. In mesenteric lymph nodes, all OLM formulations had no significant difference on uptake, indicating the absorption enhancement of OLM formulations is irrelated to the lymphatic uptake.

## 4. Discussion

Various OLM formulations incorporating lipids and surfactants have been studied. However, due to OLM’s low solubility in these excipients, previous formulations were limited by low drug loading, such as 2% in OLM micelles [28] and 0.1% in solid lipid nanoparticles [29]. Additionally, these formulations were either finished as liquids or lyophilized powders, often requiring complex preparation processes. Although OLM has a LogP of 4.31, it has low solubility in lipids and surfactants. While lipid- and surfactant-based formulation strategies are widely employed to enhance the oral absorption of water-insoluble drugs, conventional approaches such as emulsions, microemulsions, micelles, and nanoparticles are unsuitable for OLM due to these solubility limitations. In this study, we demonstrated a novel application of lipid and surfactant in a BLS to coat OLM crystals, creating solid granules with a 10% drug loading. This approach overcame the challenges of low drug loading and formulation stability. Remarkably, even though OLM remained in crystalline form, OLM MCM-TPGS solid granules significantly enhanced oral absorption, showcasing the effectiveness of this novel formulation strategy. With this approach, solubility is no longer a limiting factor when selecting lipids and surfactants, representing a significant advancement in formulation technologies, and expanding the potential applications of lipids and surfactants for water-insoluble drugs.

Ester prodrugs are commonly used to enhance solubility, permeability, and drug transport [4,30]. However, the primary criterion for these strategies to be effective is stability, which is particularly critical for oral ester prodrugs as they encounter varying pH conditions and enzymatic degradation in the GI tract. To reach systemic circulation, oral ester prodrugs must remain stable within the GI tract. OLM is a typical medoxomil ester prodrug, where the carboxylic group of OL is masked by a medoxomil ester to enhance permeability. Ideally, OLM remains intact in the GI tract and, after absorption, is converted to OL in the blood and tissues. However, OLM undergoes hydrolysis by water and esterases in the GI tract (Figure 2 and Figure 3), leading to the premature conversion of OLM to OL, loss of permeability, and consequently, low bioavailability. Because OLM exhibits pH-dependent solubility and undergoes enzymatic hydrolysis, a two-step dissolution process with esterase more accurately mimics the physiological conditions for OLM. However, most previous studies employed a one-step dissolution method, either at pH 1.2 or pH 6.8, with very few incorporating esterase into the dissolution media [21,28,29]. In this study, the addition of CE to the dissolution media effectively distinguished the differences among the OLM formulations (Figure 2C and Figure 3C), whereas all formulations showed similar release profiles in the absence of CE (Figure 2A and Figure 3A). Therefore, incorporating esterase in a two-step dissolution study is essential for accurately evaluating the dissolution behavior of ester drugs and comparing different formulations. The data also suggest that OLM may be subject to a food effect, as it achieved 100% release at pH 1.2 (fasted) (Figure 2A) but less than 3% release at pH 3.5 (fed) (Figure 3A). However, food is not currently considered a significant factor in the administration of OLM for patients. Based on the two-step dissolution data, the potential food effect may be another contributing factor to OLM’s low bioavailability.

We designed three different OLM granules using various lipids. Among these, OLM MCM-TPGS granules demonstrated our hypothesis that coating OLM crystals with a BLS increases OLM solubility and protects it from aqueous and CE-mediated degradation. OLM MCM-TPGS granules significantly increased drug release and protected OLM from CE-mediated degradation in two-step dissolution studies (Figure 2C and Figure 3C). PK studies confirmed that OLM MCM-TPGS granules enhanced oral absorption by 145% compared to OLM powder (Table 3). OLM has been solubilized in self-microemulsifying drug delivery systems (SMEDDS) and solid lipid nanoparticles, with relative bioavailability increases of 171% [21] and 232% [29], respectively, compared to OLM powder. In comparison, the relative bioavailability of OLM MCM-TPGS granules was 245% relative to OLM powder. Notably, although OLM remained in its crystalline form in the MCM-TPGS granules, the granules achieved higher bioavailability than the previously reported formulations where OLM was solubilized. This highlights the effectiveness of this novel strategy in enhancing the oral absorption of ester prodrugs.

**Figure 6:**
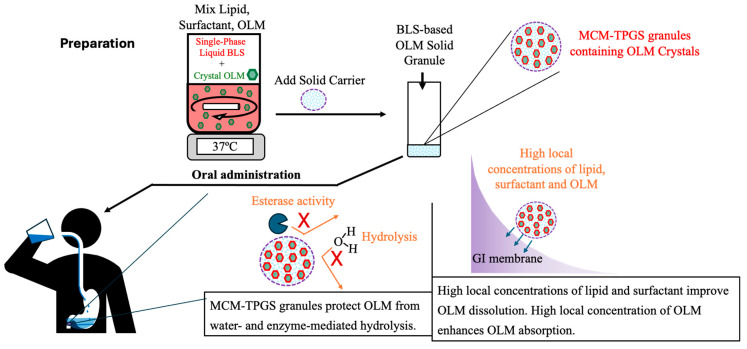
The preparation procedure and illustration of proposed mechanisms of absorption enhancement of OLM MCM-TPGS granules. The preparation of BLS-coated OLM granules is simple and scalable by using conventional mixing equipment. Following oral administration, the MCM-TPGS granules can protect crystalline OLM from water- and enzyme-mediated hydrolysis. In addition, when OLM MCM-TPGS granules reach the GI membrane, MCM C8 and TPGS are released from the solid granules, generating high local concentrations of lipid and surfactant that promote OLM dissolution. Furthermore, the high local concentration of OLM enhances OLM absorption.

Detailed analysis of the effects of lipid composition, TPGS, and the solid granule format revealed the importance of the BLS composition and solid form. OLM MCM granules failed to protect OLM from CE-mediated degradation (Figure 2C and Figure 3C) and did not enhance oral absorption (Table 3). Similarly, substituting Capmul MCM C8 with Miglyol 812 or Capmul PG-8 did not improve oral absorption (Table 3). Moreover, the liquid BLS formulation without Aeroperl 300, composed of Capmul MCM C8 and TPGS, also failed to enhance oral absorption (Table 3). Capmul MCM C8, composed of mono- and diglycerides, directly enters the bloodstream or undergoes minor digestion to free fatty acids by esterases in the small intestine. In contrast, Miglyol 812, a triglyceride, is extensively digested in the small intestine by esterases into diglycerides, monoglycerides, and eventually free fatty acids. Thus, Capmul MCM C8 is more resistant to CE-mediated degradation compared to Miglyol 812. Capmul PG-8, a propylene glycol derivative, is absorbed through the GI tract and metabolized in the liver. In two-step dissolution studies (Figure 3C), both Capmul MCM C8 and Capmul PG-8 demonstrated protection against CE-mediated hydrolysis. However, Miglyol 812 was rapidly digested by CE, causing the BLS coating composed of Miglyol 812 and TPGS to break down quickly, providing no protective effect (Figure 3C,D). Although OLM PG8-TPGS LSG and OLM MCM-TPGS LSG showed similar release profiles (Figure 3A,C), Capmul MCM C8 exhibited higher lipophilicity and has potential to increase permeability [31], thereby leading to 2-fold increase in oral absorption (Table 3). An in vitro-in vivo correlation (IVIVC) could not be established for OLM LSG, consistent with prior reports that IVIVC for lipid-based formulations is difficult due to their complex, digestion-dependent absorption [32]. We also observed potential food effects for OLM, which further complicate IVIVC. To better evaluate IVIVC for OLM LSG, future dissolution testing should incorporate lipases and relevant food components in the dissolution medium. It is known that long-chain triglycerides or fatty acids increase lymphatic drug uptake through chylomicron formation, whereas median-chain glycerides are rapidly hydrolyzed and facilitate drug absorption directly into the blood circulation [33,34]. Capmul MCM C8 is a median-chain glyceride. As shown in our results, OLM MCM-TPGS granules increased plasma and tissue uptake (Figure 5) but did not increase the lymphatic uptake. This indicates that the oral bioavailability improvement achieved with OLM MCM-TPGS granules primarily arises from enhanced dissolution and protection against both aqueous and CE-mediated degradation. The proposed mechanisms are illustrated in Figure 6. After oral administration, the coating of BLS protects OLM from water- and enzyme-mediated hydrolysis. When BLS-based OLM granules reach the GI membrane, MCM C8 and TPGS are released from the granules, generating high local concentrations of MCM C8 and TPGS that enhance OLM dissolution. Because OLM is released in a specific site, its local concentration increases, thereby facilitating OLM absorption.

OLM represents the first ester prodrug we have investigated using this coating strategy to improve oral absorption of crystalline drug forms. By formulating drug crystals into solid granules, this approach effectively addresses two challenges of lipid-based formulations such as low drug loading and poor stability. Importantly, the BLS-based granules use FDA-approved excipients, and the manufacturing process is simple, scalable and compatible with conventional pharmaceutical equipment (Figure 6). Given that many marketed ester prodrugs have poor bioavailability due to premature hydrolysis, the strategy presented here offers a generalizable formulation-based solution to prevent both water- and enzyme-mediated degradation while simultaneously improving solubility. Rather than modifying the chemical structures of ester prodrugs, this approach leverages formulation design to overcome these limitations. Thus, BLS-based formulation technology shows strong promise for advancing clinical use of ester prodrugs, potentially enabling more effective and reliable oral therapies.

## 5. Conclusions

By carefully selecting the components of the BLS, we developed and established a novel strategy of coating OLM crystals with a BLS composed of Capmul MCM C8 and TPGS in solid granule form to enhance oral absorption. The BLS coating and solid granule format not only facilitated drug release but also protected OLM from hydrolysis by water and esterases. This innovative approach does not require complete solubilization of the drug in the formulation, thereby increasing drug loading capacity. The solid granules were prepared using simple and scalable procedures, resulting in improved formulation stability. OLM MCM-TPGS granules increased bioavailability by 145% compared to OLM powder and enhanced tissue uptake. For future studies, it will be important to evaluate the stability of BLS-coated drug granules in terms of the physical state of drug crystals, lipid stability, and compatibility of excipients and drug during long-term storage at room temperature or under topical conditions. In addition, it remains unclear whether the strategy reported here is applicable to other ester prodrugs. Therefore, additional studies could investigate other ester-based prodrugs that suffer from premature hydrolysis using the approach described in this work. Overall, this novel formulation strategy holds significant potential for improving the oral absorption of ester prodrugs.

## Figures and Tables

**Table 1:** Chemical compositions of lipids used in the studies.

Lipids	Backbone	% Caproic (C6)	% Caprylic (C8)	% Capric (C10)	% Lauric (C12)	% Monoester	% Diester	% Triester	Form
Capmul MCM C8	Glycerol	0	~83	~17	0	~60	~34	~6	Liquid
Miglyol 812	Glycerol	<2	50–80	20–50	<2	0	0	100	Liquid
Capmul PG-8	Propylene Glycol	0	90	3.0	3.0	90	10	0	Liquid

Note: The pharmaceutical lipids are mixtures of each component. The percentage in the table is the mean of each component.

**Table 2:** Compositions of OLM formulations used in the studies.

Formulations	OLM (mg)	Avicel 105 (mg)	Capmul MCM C8 (mg)	Miglyol 812 (mg)	Capmul PG-8 (mg)	TPGS (mg)	Aeroperl 300 (mg)	% Drug Loading
OLM powder	9	81	-	-	-	-	-	10
OLM MCM-TPGS granules	9	-	36	-	-	18	27	10
OLM Miglyol-TPGS granules	10	-	-	30	-	30	30	10
OLM MCM granules	9	-	72	-	-	-	54	13
OLM PG8-TPGS granules	9	-	-	-	36	18	27	10
OLM MCM-TPGS liquid	9	-	36	-	-	18	-	14

**Table 3:** The mean pharmacokinetic parameters (± standard deviation) of OL in plasma after oral administration at a dose of 2 mg/kg OLM from OLM powder, OLM MCM-TPGS granules, OLM MCM-TPGS Liquid, Miglyol-TPGS granules, OLM MCM granules and OLM PG8-TPGS granules to rats, respectively, using WinNonlin Phoenix (version 8.40).

Parameter	OLM Powder (*n* = 6)	OLM MCM-TPGS Granule (*n* = 4)	OLM MCM-TPGS Liquid (*n* = 5)	OLM M812-TPGS Granule (*n* = 5)	OLM MCM Granule (*n* = 4)	OLM PG8-TPGS Granule (*n* = 5)
*C*_max_ (ng/mL)	67.0 ± 20.8	167 ± 27.5 ***	96.0 ± 29.5 ^##^	61.1 ± 28.9 ^###^	51.6 ± 20.5 ^###^	76.6 ± 35.6 ^###^
T_1/2_ (h)	4.61 ± 1.02	13.5 ± 20.3	5.91 ± 1.58	4.26 ± 1.39	7.31 ± 4.77	4.61 ± 3.20
*T*_max_ (h)	2.00 ± 0.00	1.75 ± 0.500	1.60 ± 0.548	2.00 ± 0.00	1.75 ± 0.500	1.80 ± 0.447
AUC_last_ (ng·h/mL)	341 ± 93.1	700 ± 107 **	509 ± 225	329 ± 115 ^##^	262 ± 87.1 ^##^	357 ± 109 ^##^
AUC_inf_ (ng·h/mL)	347 ± 90.9	851 ± 390 **	529 ± 232	341 ± 119 ^##^	281 ± 82.3 ^##^	372 ± 114 ^#^
*V*_z_/F	43.7 ± 27.7	33.2 ± 36.8	38.5 ± 21.1	37.8 ± 16.0	79.2 ± 51.2	35.2 ± 20.4
CL/F	6.24 ± 2.23	2.65 ± 0.871	4.41 ± 1.86	6.38 ± 1.93	7.55 ± 1.94 ^#^	5.82 ± 1.80
Relative bioavailability ^a^	-	245%	152%	98%	81%	107%

^a^ Relative bioavailability was calculated by comparing OLM formulations with OLM powder. *C*_max_, maximum plasma concentration; *T*_max_, time to peak concentration; AUC_last_, area under the plasma concentration-time curve from time 0 to the last measurable concentration; AUC_inf_, area under the plasma concentration-time curve from time 0 to infinity; *V*_z_/F, apparent volume of distribution; CL/F, apparent total body clearance; ** *p* < 0.01 and *** *p* < 0.001: Significant difference compared to OLM Powder. ^#^ *p* < 0.05, ^##^ *p* < 0.01, and ^###^ *p* < 0.001: Significant difference compared to OLM MCM-TPGS granule. The results are present as mean and standard deviation. The groups were compared by one-way ANOVA followed by Tukey’s post hoc test.

## Data Availability

Dataset available on request from the authors.
